# Role of p53 Serine 46 in p53 Target Gene Regulation

**DOI:** 10.1371/journal.pone.0017574

**Published:** 2011-03-04

**Authors:** Leonie Smeenk, Simon J. van Heeringen, Max Koeppel, Bianca Gilbert, Eva Janssen-Megens, Hendrik G. Stunnenberg, Marion Lohrum

**Affiliations:** 1 Department of Molecular Biology, Faculty of Science, Nijmegen Centre for Molecular Life Sciences, Radboud University Nijmegen, Nijmegen, The Netherlands; 2 Georg-Speyer-Haus, Frankfurt, Germany; University of Illinois at Chicago, United States of America

## Abstract

The tumor suppressor p53 plays a crucial role in cellular growth control inducing a plethora of different response pathways. The molecular mechanisms that discriminate between the distinct p53-responses have remained largely elusive. Here, we have analyzed the p53-regulated pathways induced by Actinomycin D and Etoposide treatment resulting in more growth arrested versus apoptotic cells respectively. We found that the genome-wide p53 DNA-binding patterns are almost identical upon both treatments notwithstanding transcriptional differences that we observed in global transcriptome analysis. To assess the role of post-translational modifications in target gene choice and activation we investigated the genome-wide level of phosphorylation of Serine 46 of p53 bound to DNA (p53-pS46) and of Serine 15 (p53-pS15). Interestingly, the extent of S46 phosphorylation of p53 bound to DNA is considerably higher in cells directed towards apoptosis while the degree of phosphorylation at S15 remains highly similar. Moreover, our data suggest that following different chemotherapeutical treatments, the amount of chromatin-associated p53 phosphorylated at S46 but not at pS15 is higher on certain apoptosis related target genes. Our data provide evidence that cell fate decisions are not made primarily on the level of general p53 DNA-binding and that post-translationally modified p53 can have distinct DNA-binding characteristics.

## Introduction

The tumor suppressor p53 plays a central role in response to cellular stress such as DNA damage. In a wide variety of human cancers the pathways leading to growth arrest or apoptosis are disrupted. This highly correlates with p53 mutations, especially in the DNA-binding domain [1]. In response to a cellular stress signal p53 gets stabilized and regulates the expression of target genes involved in growth arrest, apoptosis and other responses [2].

An important question for the p53 research is whether and how p53 discriminates between target genes to be activated or repressed, resulting in a particular cellular outcome. Several models have been proposed to explain how p53 determines the cellular outcome. Several lines of evidence lead to the threshold model in which the amount of p53 protein present in a cell determines if cells go into apoptosis [3]. Other models have been described in which co-factors, p53-binding factors and post-translational modifications play an important role in p53 target gene selection [4]. In the selective binding model, selectivity of target gene activation and repression takes place at the level of DNA-binding. In the selective context model, p53 is thought to first bind to all accessible sites in the genome after which other determinants like p53-binding factors and the presence of p53 PTMs determine the cellular outcome. Yet, it remains unresolved which of these models of p53 target gene choice best reflects the actual *in vivo* situation upon different stress signals.

The p53 activity is tightly regulated in the cell by co-factors [5] as well as post-translational modifications [6]. Among the p53-binding proteins that are described to influence the cellular outcome are the apoptosis-stimulating proteins of p53 (ASPP) proteins that interact with p53 and specifically stimulate p53-induced apoptosis but not cell cycle arrest and iASPP that inhibits p53-mediated apoptosis [7]. The Hematopoietic Zinc Finger protein (HZF) that is induced by p53 and binds to its DNA-binding domain, facilitates p53-binding to cell cycle arrest target genes resulting in preferential cell cycle arrest [8]. Also the human cellular apoptosis susceptibility protein (hCAS/CSE1L) has been shown to influence the p53-mediated transactivation by binding to a subset of p53 target genes [9].

A series of post-translational modifications of p53 is involved in mediating transactivation upon stress by stabilizing and activating p53, such as phosphorylation, acetylation, methylation, neddylation, sumoylation and ubiquitination [6], [10], [11]. Besides stabilization and activation, several post-translational modifications are thought to play a role in target gene selectivity [3], [4], [10]. Numerous Serine and Threonine residues of p53 are targets for phosphorylation. Some amino acids that are phosphorylated upon stress lead to a general stabilization and activation of p53, such as Serine 15, which is phosphorylated in an ATM-dependent manner [12]. Phosphorylation of Serine 46, on the other hand, is proposed to be involved in the selectivity of apoptotic target genes, such as the p53 apoptosis inducing protein 1 (p53AIP1) in response to DNA damage [13]. This phosphorylation site can be regulated by several kinases, by e.g. homeodomain interacting protein kinase 2 (HIPK2) [14], [15], dual-specificity tyrosine-phosphorylation-regulated kinase 2 (DYRK2) [16], ataxia-telangiectasia mutated (ATM) [17], protein kinase C δ (PKCδ) [18], AMP-activated protein kinase catalytic subunit α (AMPKα) [19] or p38 mitogen-activated protein kinase (p38 MAPK) [20]. The fact that several kinases can phosphorylate Serine 46 suggests that this may be a very important modification for the regulation and function of p53. One of the most interesting current research questions is the exact contribution of this phosphorylation site to the selectivity of the global transcriptional program of p53.

To investigate how the selectivity of p53 target genes is globally mediated, we have performed genome-wide DNA-binding and expression analysis upon different chemotherapeutic treatments. We can show that different treatments induce the transcriptional activation or repression of treatment specific sets of target genes while p53 binds indiscriminately to a general pool of p53-binding sites. Importantly, we found that the extent to which chromatin associated p53 is phosphorylated at Serine 46 increases significantly upon apoptosis-inducing Etoposide treatment whereas the amount of DNA-bound p53 that is phosphorylated at Serine 15 remains similar upon both treatments. Finally, we observed specific differences of binding of p53 phosphorylated at Serine 46 to several direct transcriptional target genes involved in apoptosis induction.

## Results

### DNA-binding of p53 upon chemotherapeutic treatment

**Figure 1:**
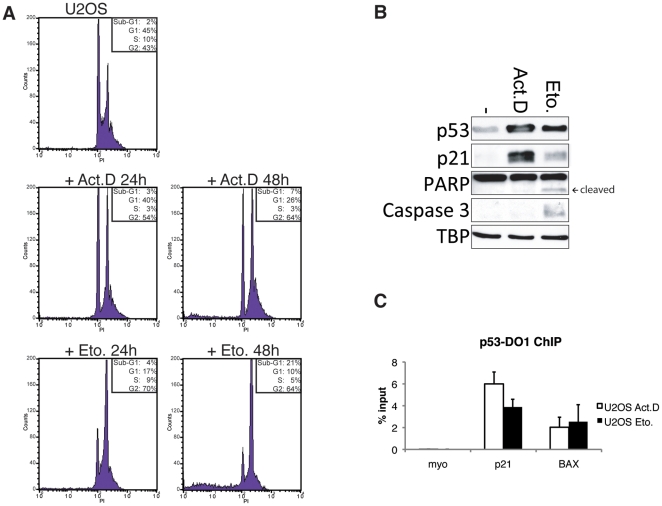
Treatment of U2OS cells with Actinomycin D or Etoposide activates p53. (A) Representative cell cycle profiles of U2OS cells untreated or treated for 24 or 48 hours with 5 nM Actinomycin D or 10 µM Etoposide. (B) Western blot showing p53, and p21, cleaved PARP and active Caspase-3 protein levels in whole cell extracts of U2OS cells untreated or treated for 24 hours with 5 nM Actinomycin D or 10 µM Etoposide. TBP is used as loading control. (C) ChIP recovery of p53-binding to the p21 and BAX promoter in U2OS cells treated for 24 hours with 5 nM Actinomycin D or 10 µM Etoposide. ChIP was performed with a p53-antibody (p53-DO1) and qPCR analysis was performed with primers for the respective binding sites. Binding to Myoglobin (myo) was used as a negative control. Error bars represent standard deviation of three individual experiments.

In order to gain more insight into the molecular basis of p53-mediated growth arrest versus apoptosis induction, we studied the transcriptional pathways activated by p53 upon two different cellular fates. Therefore, we treated U2OS osteosarcoma cells expressing wild type p53 with two chemotherapeutic reagents, Actinomycin D to induce cell cycle arrest and Etoposide to increase the apoptotic cell population (Figure 1A). Treatment with Etoposide resulted in PARP cleavage and active Caspase 3 (Figure 1B), indicating that the cells are going into apoptosis, whereas cells treated with Actinomycin D did not. Upon both treatments p53 is stabilized resulting in p21-induction (Figure 1B). To study the transcriptional responses upon the treatments, p53 chromatin-immunoprecipitations (ChIPs) were performed to test for p53-binding to the promoters of the cell cycle inhibitor p21 and the pro-apoptotic target gene BAX. Upon both treatments the cell-cycle arrest as well as the pro-apoptotic target genes were bound by p53 as shown in specific chromatin-immunoprecipitation experiments (Figure 1C), whereas chromatin-immunoprecipitations with IgG instead of a specific antibody did not result in a specific enrichment of target sites (Figure S1A). Thus, the question arises how under different cell fate decisions the respective target gene specificity is regulated. To obtain insight into this question, we analyzed the global DNA binding sites of p53 under both conditions using ChIP-sequencing (ChIP-seq). We identified 2,132 p53-binding sites upon Actinomycin D treatment and 2,920 upon Etoposide treatment (Table 1) using the MACS peak recognition [21]. Identified p53-binding sites were annotated and the locations of p53-binding sites were assigned to RefSeq genes [22].

**Table 1:** Sequenced reads of p53-DO1 ChIP-seq.

		Actinomycin D	Etoposide
**Reads**	Uniquely mapped reads (millions)	5.4	6.1
	Normalized reads (millions)	5.3	5.3
**Peaks**	Number of peaks	2,132	2,920
	Average height	35	35
	Minimum height	8	8

**Figure 2:**
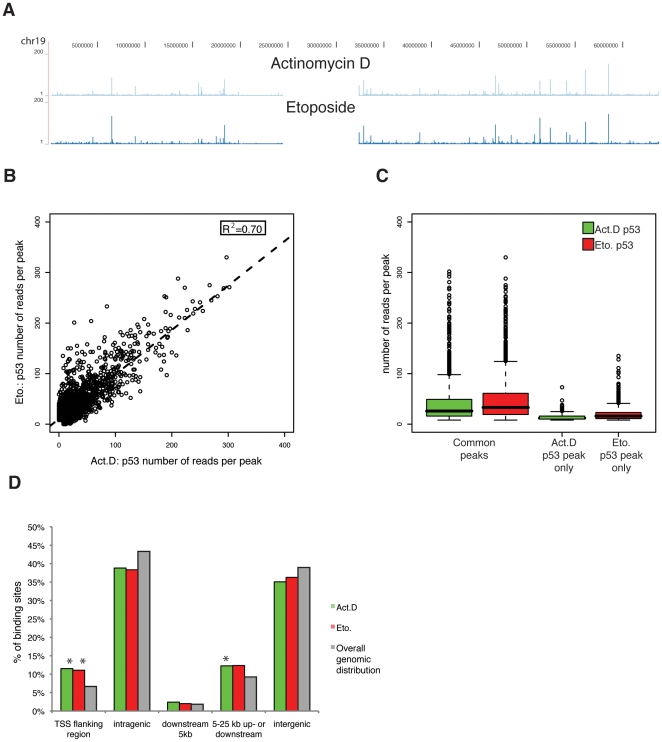
Genome-wide p53-binding profiles. (A) Representative genomic overview of p53-binding to chromosome 19 upon 5 nM Actinomycin D or 10 µM Etoposide treatment for 24 h as determined by ChIP-Seq using the Genome analyzer (Illumina) and visualization using the UCSC genome browser. (B) The number of reads per peak (RPP) of p53-binding upon Actinomycin D is plotted versus the RPP of p53-binding upon Etoposide treatment. To determine the correlation between the RPPs of both treatments the square of the sample correlation coefficient between both treatments was calculated (R^2^ = 0.70). (C) The average number of reads per peak (RPP) for the common p53 peaks as well as the treatment preferential peaks upon Actinomycin D or Etoposide treatment are visualized in a boxplot. (D) Genome-wide distribution of the p53-binding sites relative to RefSeq genes. Locations of binding sites are divided in Transcriptional Start Site (TSS) flanking region (5 kb upstream of TSS+first exon+first intron), intragenic region (all introns and exons except first), 5 kb downstream (5 kb downstream of last exon), 5–25 kb up- or downstream of a RefSeq gene or intergenic regions (everything else). The genomic distribution is defined as the number of nucleotides per region divided by the total number of nucleotides in the genome. The asterisk represents significant enrichment compared to overall genomic distribution.

Surprisingly, most DNA-binding sites of p53 were present upon both treatments (Figure 2A). Using strict peak settings, as many as 76% of the peaks detected in Actinomycin D treated cells are also bound by p53 upon Etoposide (Table 2). The similarity in binding occupancy following either treatment is further substantiated when the number of reads per peak (RPP) for Actinomycin D versus Etoposide is plotted (Figure 2B), which shows a good correlation of the RPP between both treatments (R^2^ = 0.70). Furthermore, the common binding sites are the most prevalent peaks as shown by a median number of reads per peak (RPP) of 26 and 33 for Actinomycin D and Etoposide treatment respectively, while the peaks preferential for Actinomycin D or Etoposide were significantly lower than the common peaks with a median RPP of 12 (Actinomycin D, P<0.0001, two-sample Kolmogorov–Smirnov test) and 16 (Etoposide, P<0.0001) (Figure 2C). Additionally, the number of reads detected in ‘the respective other treatment’ is still significantly higher than background (P<0.0001, Figure S1B), but lower than peak-threshold and therefore not detected as common peaks. Thus, the low, preferentially detected binding sites are most likely also occupied by p53 upon the respective other treatment but fall just under the threshold in that treatment. Decreasing the threshold for peak detection includes these peaks, but would at the same time increase the number of false positives detected. Therefore, we chose to use a strict threshold to detect and subsequently analyze only high-confidence p53 peaks. Upon both treatments the identified peaks are significantly enriched at transcriptional start site (TSS) flanking regions encompassing promoters as well as the broader promoter-enhancer regions, 5–25 kb up- or downstream from the respective genes (Figure 2D). To characterize sequences underlying the binding sites, we analyzed them with the p53scan algorithm [23] and found that the vast majority of the identified p53-binding sites (86% and 88% respectively) contain a p53 consensus motif irrespective of the treatment. Comparing our binding data upon Actinomycin D treatment or Etoposide treatment with another available global p53 binding set, the data set from Wei *et al.* [24], we found that 72% and 81% respectively of the PET3+ binding sites from Wei *et al.* were bound in our p53 binding set upon Actinomycin D or Etoposide treatment, indicating a high overlap irrespective of cell line or treatment used.

**Table 2:** Overlap of p53-binding sites.

	Binding sites
**Actinomycin D**	2,132
**Etoposide**	2,920
**Overlap**	1,612

Thus, upon two different chemotherapeutic treatments we found very similar overall genome-wide binding sites of p53. We hypothesize that the general p53-binding patterns that overlap to a large extent are unlikely to fully account for the biological differences observed upon these two treatments in U2OS cells.

### Expression analysis upon Actinomycin D and Etoposide treatment

**Figure 3:**
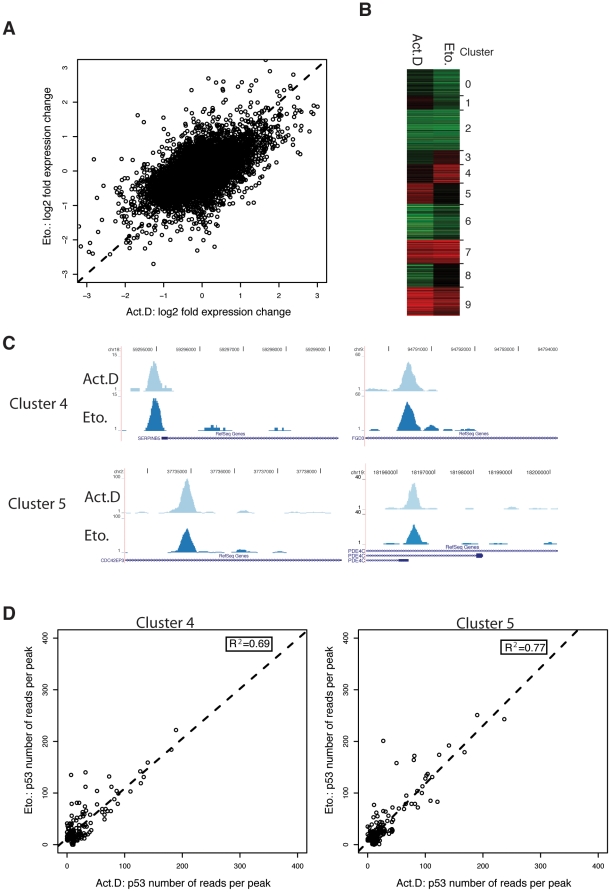
Global expression analysis upon Actinomycin D and Etoposide treatment. (A) Global expression analysis of U2OS cells treated for 24 hours with Actinomycin D or Etoposide was performed using Affymetrix exon arrays. The ratio of expression changes (log2 signal intensity 24 hours/0 hours) upon Actinomycin D treatment versus Etoposide treatment was plotted against each other. (B) K-Means clustering results of the expression change of genes with a p53-binding site within the transcript or 25 kb up- or downstream upon Actinomycin D or Etoposide treatment. Upregulation is indicated in red; downregulation in green. (C) p53-binding as determined by ChIP-Seq visualized using the UCSC Genome Browser to two genes which are preferentially upregulated upon Etoposide (cluster 4) and two genes which are preferentially upregulated upon Actinomycin D treatment (cluster 5). (D) The number of reads per peak (RPP) of p53-binding upon Actinomycin D is plotted versus the RPP of p53-binding upon Etoposide for the target genes of cluster 4 (left) and cluster 5 (right). To determine the correlation between the RPP of both treatments the square of the sample correlation coefficient between both treatments was calculated (R^2^ = 0.69 and R^2^ = 0.77 respectively).

To study the effect of Actinomycin D or Etoposide treatment on the transcriptome level, we analyzed respective expression changes by performing microarray analysis experiments on Affymetrix Human Exon Arrays (Affymetrix, Santa Clara, CA). Analyzing the expression changes, we found that treatment of U2OS cells with either Actinomycin D or Etoposide resulted in treatment-specific expression patterns as demonstrated in the plot showing ratios of gene expression changes upon Actinomycin D treatment versus Etoposide treatment (Figure 3A). The correlation between both treatments is 0.36, which is significantly lower than the correlation for p53 binding between the two treatments which is 0.70. Thus, many genes are preferentially up- or down-regulated upon one of the two treatments.

To analyze the relationship between target genes that were bound by p53 and target genes changing expression upon treatment, we correlated the binding with the expression data (Affymetrix core transcripts). In total, upon Actinomycin D treatment 1320 genes have a p53-binding site in close proximity i.e. within the gene body or 25 kb up- or downstream of a gene, of which 185 (14%) changed at least 1.7 fold in expression (Table S1) and upon Etoposide treatment 1,710 genes have a p53-binding site in close proximity of which 177 (10%) changed at least 1.7 fold (Table S2). To analyze whether high expression changes also correlate with stronger binding in the ChIP-seq data we plotted the RPP for p53 versus the expression change. We observed no significant correlation between the number of reads in the ChIP-seq experiments and change in expression in either treatment, neither for up- nor for down-regulated transcripts (Figure S2). To further study the relationship between target genes which have a p53-binding site nearby and expression changes, we clustered genes that change in their level of expression and have a p53-binding site using a k-Means algorithm based on uncentered correlation of the expression change (Figure 3B). We observed gene clusters that were clearly differentially regulated only upon one of the two treatments, such as target gene cluster 4 and 5 (Table S3). Interestingly, we found that genes which are preferentially upregulated upon either Etoposide (cluster 4) or Actinomycin D treatment (cluster 5) are nonetheless indiscriminately bound by p53 upon both treatments as shown for the indicated examples of the SerpinB5 and FGD3 genes for cluster 4 and the CDC42EP3 and PDE4C genes for cluster 5 (Figure 3C). Comparing the RPP between the two treatments of the cluster 4 or 5 target genes shows that the target genes of these two clusters, highly correlate with respect to their p53 DNA binding (Figure 3D), although they show differential expression upon the two treatments. Thus, our data show that differentially expressed target genes can be bound by p53 indiscriminately upon both treatments.

### Differential phosphorylation of chromatin associated p53 upon chemotherapeutic treatment

**Figure 4:**
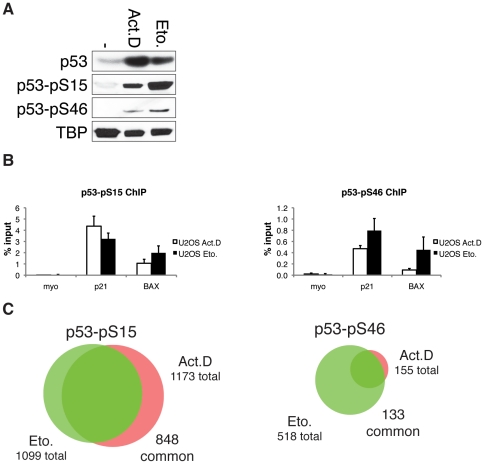
DNA-binding of p53 phosphorylated at Serine 15 and 46. (A) Western blot showing protein levels of p53, p53 phosphorylated at Serine 15 (p53-pS15) and p53 phosphorylated at Serine 46 (p53-pS46) in whole cell lysates of U2OS cells untreated or treated for 24 hours with 5 nM Actinomycin D or 10 µM Etoposide. TBP is shown as loading control. (B) ChIP-qPCR recovery of p53-pS15 and p53-pS46 binding to the p21 and BAX promoter in U2OS cells treated for 24 hours with 5 nM Actinomycin D or 10 µM Etoposide. ChIP was performed with p53-pS15-antibody or p53-pS46-antibody and qPCR analysis was performed with primers for respective binding sites. Myoglobin (myo) was used as a negative control. Error bars represent standard deviation of three individual experiments. (C) Overlap of p53-pS46 binding sites (left panel) and p53-pS15 binding sites (right panel) of ChIP-seq performed after 5 nM Actinomycin D or 10 µM Etoposide treatment for 24 h using the Genome analyzer (Illumina).

Since overall binding patterns do not appear to account for the selective cellular responses to different treatments, we investigated whether p53 post-translational modifications could be involved in the cellular response decision. We set out to investigate the role of p53 phosphorylated at Serine 46 (S46) in comparison to p53 phosphorylated at Serine 15 (S15), a post-translational modification that is thought to activate and stabilize p53 independently of specific stress signals. Therefore, we analyzed global DNA-binding of p53 phosphorylated at Serine 46 and 15 by ChIP-sequencing. We detected p53 phosphorylated at S15 and S46 upon both treatments in whole cell lysates (Figure 4A). In ChIP-qPCR experiments we consistently observed a statistically significant difference in binding of the phosphorylated p53 forms to the BAX promoter (Figure 4B). In Etoposide treated U2OS cells, about five times more p53-pS46 is bound to the BAX promoter as compared to Actinomycin D treated cells. Since BAX is an apoptotic target gene we assessed whether the higher degree of DNA-binding of p53 phosphorylated at Serine 46 upon Etoposide treatment is a general feature of apoptotic target genes. Therefore, we analyzed the genome-wide DNA binding sites bound by p53 in either Actinomycin D or Etoposide treatment for binding of p53 phosphorylated at S46 and of p53 phosphorylated at S15 upon both treatments by performing ChIP-seq (Table 3). Most strikingly, apoptosis-inducing Etoposide treatment appears to significantly increase the overall S46 phosphorylation state of p53 bound to chromatin. The number of genomic locations occupied by p53 phosphorylated at S46 is ∼5.5 times higher upon Etoposide treatment than upon Actinomycin D treatment (Figure 4C). For p53 phosphorylation at S15 on the other hand, we found a similar number of bound regions and most of the binding sites are bound by p53 phosphorylated at S15 upon both treatments (Figure 4C and Table 3). Notably, of all p53 DNA binding sites about 45% (for Actinomycin D treatment) and 37% (for Etoposide treatment) are bound by phosphorylated p53 (Figure S3). The p53-pS46 subset of binding sites is almost entirely included in the subset of p53-pS15 upon both treatments suggesting that all binding sites bound by p53-pS46 are bound by p53-pS15 as well or the bound proteins are phosphorylated at both S15 and S46 residues (Figure S3).

**Table 3:** Sequenced reads of p53-pS15 and p53-pS46 ChIP-seq.

		Uniquely mapped reads (millions)	Normalized reads (millions)	Number of peaks overlapping with DO1	Average height	Minimum height
**Actinomycin D**	p53-pS15	8.2	5.3	1,173	20	6
	p53-pS46	5.3	5.3	140	16	7
**Etoposide**	p53-pS15	8.7	5.3	1,099	24	6
	p53-pS46	6.8	5.3	518	20	6

**Figure 5:**
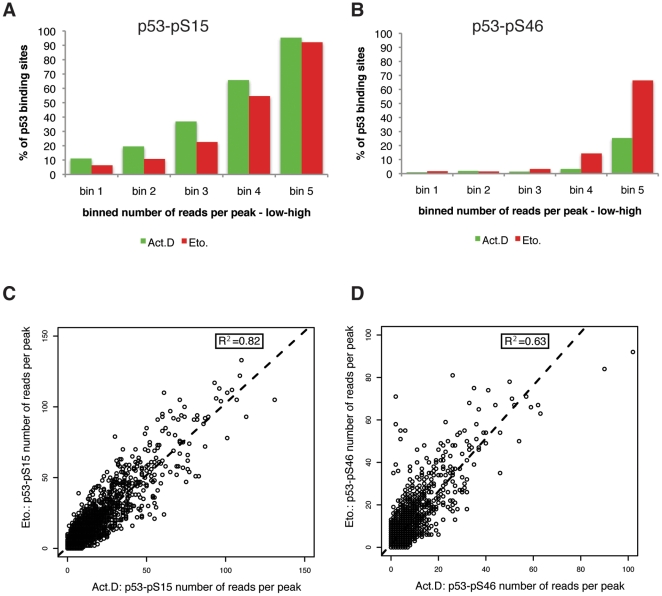
Analysis of genome-wide binding profiles of phosphorylated p53. (A–B) Percentage of overall p53-binding sites as obtained from ChIP-seq that are enriched for binding of phosphorylated p53, p53-pS15 or p53-pS46, relative to peak strength. The identified overall p53-binding sites were ranked according to the number of reads per peak and divided into five groups ranking from low to high. For each bin the percentage of p53-binding sites containing also a peak for p53-pS15 (A) or p53-pS46 (B) was plotted. (C–D) Number of reads per peak (RPP) of p53-pS15 (C) and p53-pS46 binding (D) in Actinomycin D versus Etoposide treated cells. To determine the correlation between Actinomycin D versus Etoposide RPP the square of the sample correlation coefficient between both treatments for p53-pS15 (R^2^ = 0.82) and p53-pS46 (R^2^ = 0.63) was calculated.

To examine whether there is a correlation between p53 binding strength and phosphorylation status of bound p53, we divided the general p53-DO1 binding sites into five subgroups, from low (bin 1) to high (bin 5) total number of reads per peak (RPP), and plotted the fraction of peaks with either modification in each group (Figure 5A and B). Strikingly, for Etoposide treated cells nearly 70% of the strongest bound sites (bin 5) are bound by p53-pS46, whereas upon Actinomycin D treatment only 25% of these sites were bound by p53-pS46 (Figure 5B). For p53-pS15, on the other hand, we could not detect this difference, this modification was equally found within the highest bound sites (Fig. 5A). Thus, we observed that upon Etoposide treatment significantly more of p53 bound to strong binding sites is phosphorylated at S46 than upon Actinomycin D treatment. For a direct comparison of the post-translationally modified p53 and its DNA binding upon the two different treatments, we plotted the RPP for p53-pS15 and p53-pS46 upon Actinomycin D versus Etoposide treatment (Figure 5C and D). For p53-pS15, the RPP correlates well between both treatments (R^2^ of 0.82), implying p53 phosphorylated at S15 is bound to similar sites with comparable strength upon both treatments (Figure 5C). On the other hand, the correlation for p53-pS46 between both treatments is considerably lower (R^2^ of 0.63) (Figure 5D). Thus, there are numerous target sites that upon Etoposide treatment are bound by p53 that is phosphorylated at S46 to a higher extent than upon Actinomycin D treatment.

Among those target genes that are differentially bound by p53 phosphorylated at S46 are also the two known apoptosis related target genes BAX and PUMA. We found that for these apoptotic target genes BAX and PUMA binding of p53 phosphorylated at S46 was more than 2-fold higher upon Etoposide treatment than upon Actinomycin D treatment (Figure S4). This difference is not found for the two cell cycle related target genes, p21 and MDM2 upon Actinomycin D and Etoposide treatment (Figure S4). p21 and MDM2 show comparable binding of phosphorylated p53 upon both treatments. Interestingly, the extent of DNA-bound p53 that is phosphorylated at pS15 was similar between all four target genes (Figure S4).

### Functional annotation of target genes bound by p53 phosphorylated at S46

We performed a functional annotation clustering using DAVID [25] of target genes that were bound by p53 phosphorylated at S46 to analyze their potential biological roles. Upon Etoposide treatment several functional clusters contain significantly enriched sets of p53 target genes (corrected P-value (Benjamini)<0.01) (Table 4), whereas upon Actinomycin D treatment, no set of target genes involved in a specific biological category is significantly enriched (data not shown). The most enriched cluster upon Etoposide treatment is the p53-signaling pathway, followed by the functional categories of nuclear envelope and cell cycle regulation (Table 4). Interestingly, several genes involved in apoptosis are also present (cluster 15, Table 4). Among those are well known p53 apoptotic target genes like BAX, PUMA (BBC3), BCL2L1, TRIAP1 and TNFRSF6 as well as genes which have been implicated generally with p53-signaling before like STK17A, ZMAT3, LGALS7 and BIRC8. Thus, p53 phosphorylated at S46 is bound to a broad spectrum of target genes of which the apoptosis genes constitute a specific subset.

**Table 4:** Functional clustering analysis of p53-pS46 bound RefSeq genes, ranked according to corrected P-value (Benjamini).

Annotation cluster	1^st^ Term cluster	Count	PValue	Benjamini
**1**	p53 signaling pathway	8	5.63E-05	1.13E-02
**2**	nuclear envelope	10	1.47E-04	1.20E-01
**3**	cell cycle arrest	6	1.64E-03	7.63E-01
**4**	negative regulation of biological process	28	5.44E-05	2.48E-01
**5**	regulation of anatomical structure, morphogenesis	4	1.13E-02	9.34E-01
**6**	cytoskeleton	23	4.91E-03	7.60E-01
**7**	cellular structure, morphogenesis	13	4.96E-03	9.76E-01
**8**	response to DNA damage stimulus	13	1.18E-04	2.67E-01
**9**	WW domain	3	1.69E-02	1.00E+00
**10**	cell communication	56	1.24E-02	9.28E-01
**11**	cytoskeleton	23	4.91E-03	7.60E-01
**12**	fibroblast proliferation	3	9.87E-03	9.26E-01
**13**	cation binding	52	2.36E-02	9.99E-01
**14**	release of cytochrome c from mitochondria	3	6.33E-03	9.64E-01
**15**	apoptosis	9	1.66E-02	9.21E-01

### Expression analysis of differentially bound p53-pS46 target genes

**Figure 6:**
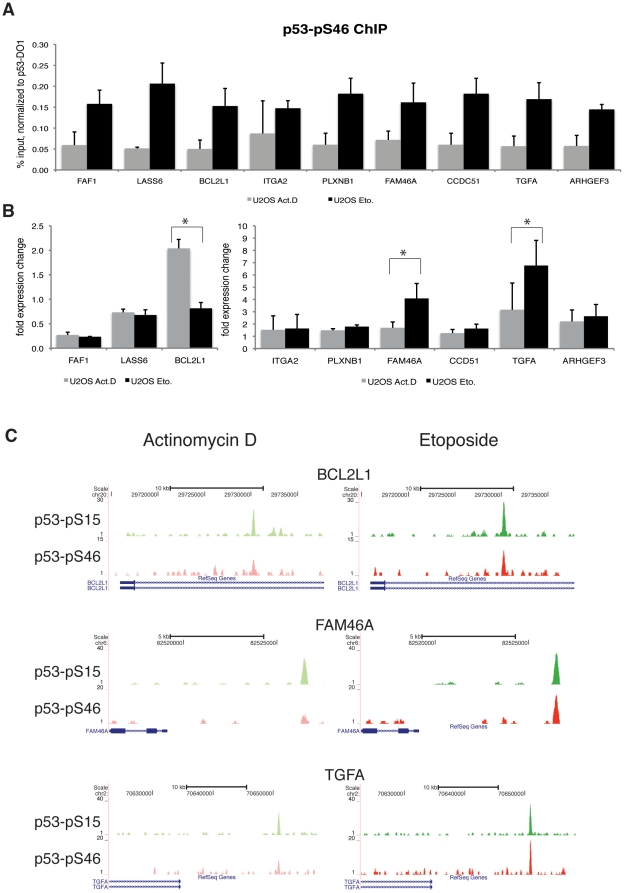
Target genes differentially bound by p53-pS46. (A) ChIP-qPCR recovery of loci which show a higher degree of bound p53 phosphorylated at S46 upon Etoposide treatment in ChIP-seq. U2OS cells were treated with Actinomycin D or Etoposide for 24 hours, before chromatin was isolated. ChIP was performed with the p53-pS46-antibody and qPCR analysis was performed with primers for the putative binding sites. Shown is the recovery of p53-pS46 normalized to the recovery of total p53-DO1 binding in Etoposide or Actinomycin D treated U2OS cells. Error bars represent standard deviation of three individual experiments. (B) Expression change of the respective target genes in U2OS cells treated with Actinomycin D or Etoposide for 24 hours. After cDNA synthesis qPCR was performed and results were normalized against GAPDH expression. Shown is fold over untreated cells. Error bars represent standard deviation of three individual experiments. Asterisks indicate statistical significance shown by Student's t-test (* = P<0.01). (C) p53-pS15 and p53-pS46 DNA-binding as determined by ChIP-Seq with the Genome analyzer (Illumina) and visualized using the UCSC genome browser. Shown are binding loci of BCL2L1, FAM46A and TGFA of Actinomycin D treated cells (left panel) and Etoposide treated cells (right panel).

We investigated in more detail those p53 binding sites showing stronger binding of p53 phosphorylated at S46 as well as an expression change upon Etoposide treatment. We identified 98 binding sites close to 94 unique genes which differ at least 1.2 fold in expression between Actinomycin D and Etoposide (Table S4). We validated a random set of these binding sites by ChIP-qPCR and monitored expression changes of the respective target genes upon Actinomycin D or Etoposide treatment by RT-qPCR. For all tested target sites the binding of p53 phosphorylated at S46 is higher upon Etoposide treatment than upon Actinomycin D treatment (Figure 6A) while the difference of binding of p53 phosphorylated at S15 is much smaller upon the two treatments (Figure S5). Testing whether the higher degree of binding of p53 phosphorylated at S46 is correlated with an expression change of the respective target genes, we detected for BCL2L1, FAM46A, and TGFA a statistically significant difference in expression change for Etoposide versus Actinomycin D treatment (Figure 6B). Analyzing the ChIP-sequencing data of those three target genes, we indeed see that while the binding of p53 phosphorylated at S15 remains almost unchanged upon both treatments, the binding of p53 phosphorylated at S46 is clearly stronger (2-fold) upon Etoposide treatment than upon Actinomycin D treatment (Figure 6C). Thus, the tested target genes show a distinctive binding pattern of p53 phosphorylated at S46 upon Etoposide treatment which in some cases is also accompanied by a respective expression change. Thus, we conclude that it is feasible that post-translational modifications of p53, in particular the phosphorylation of S46 can contribute to the p53 target gene selectivity and can therefore influence differential p53 cellular response pathways.

## Discussion

An essential issue in the present p53 research is to understand the molecular basis for the discrimination between the widely varying cellular responses upon p53 activation. The regulation mechanisms of p53's ability to selectively activate or repress a certain set of target genes have remained elusive. To explain the possible regulation mechanisms several models have been postulated like the latency model, in which p53 is in a latent state in the absence of cellular stress and becomes activated upon cellular stress [26]. In the threshold model the amount of p53 determines cellular outcome [27]. Recently, the existing lines of evidence have been incorporated into the selective binding model, in which selectivity of target gene activation and repression takes place directly at the level of DNA-binding, and the selective context model. In the selective context model p53 first binds to all accessible sites in the genome and then other determinants like post-translational modifications of p53, the presence of co-factors and chromatin architecture determine cellular outcome [4]. Most of the experimental evidence for the molecular mechanism of the p53 response has been based so far on a selective set of target genes. Therefore, we set out to gain more insight in global p53-binding and target gene activation or repression. We compared genome-wide p53 DNA-binding upon the two different chemotherapeutic treatments Actinomycin D and Etoposide by ChIP-seq and analyzed subsequent expression changes. To elucidate a possible involvement of post-translational modifications in the actual target gene selection, we assessed the role of p53-phosphorylation at Serine 15 and 46.

The overall genome-wide binding pattern of p53 is very similar between the two different treatments. A large number of binding sites are occupied by p53 upon both Actinomycin D and Etoposide treatment. Overall, only a fraction of all bound p53-target genes actually change in expression upon the particular induction. This is in line with other studies, which show that the binding of p53 to the regulatory regions of a target gene is not necessarily directly correlated to an expression change of every bound gene [24], [28], [29], [30], [31]. In contrast to the similar occupancy of binding sites, cell cycle analysis and expression profiles show a marked difference in cellular outcome. Interestingly, we found that even a subset of genes which display differential expression upon the treatments was bound by p53 to a comparable degree upon both treatments. This makes the selective binding model [4] unlikely since our obtained overall p53-binding data cannot explain the difference in transcriptional activation upon the different treatments. Thus, it is unlikely that different cellular outcomes can be explained only on the basis of selective binding of p53.

As suggested in the selective context model, additional levels of regulation appear to be necessary to mediate target gene selection, modulated by e.g. either post-translational modifications of p53, co-factors, p53-binding factors, p53 responsive microRNAs (miRNAs), p53 family members or chromatin remodeling factors. While some post-translational modifications (PTMs) are thought to be necessary for p53 activity in general such as p53 phosphorylation at Serine 15 [32], phosphorylation of Serine 46 has been proposed to be involved in the activation of certain apoptotic target genes, such as p53 apoptosis inducing protein 1 (p53AIP1) in response to DNA damage resulting in apoptosis of the cells [13], [15], [16], [33], [34], [35], [36]. To find out whether the DNA-binding of p53 phosphorylated at S46 plays a role in target gene selectivity, in particular for the selection of apoptotic target genes, we studied genome-wide DNA-binding profiles of p53 phosphorylated at S46, and analyzed the binding profiles of p53 phosphorylated at S15 in comparison. As a matter of fact, we detected a considerably higher degree of p53 phosphorylated at S46 bound to target genes upon Etoposide treatment, although binding of p53-pS46 is in principle also present in cells treated with Actinomycin D. Analyzing the bound targets in more detail, we found that the DNA-binding of p53 phosphorylated at S46 is not restricted to apoptotic target genes. Thus, the degree of DNA-bound p53 that is phosphorylated at S46 is higher upon Etoposide treatment and on a genome-wide DNA-binding scale a broad spectrum of individual target genes involved in different cellular functions can be found, including apoptotic target genes like BAX and PUMA.

Since it has been shown that p53 phosphorylated at pS46 is involved in selectivity of certain apoptotic target genes, [13], [33], [34], we investigated target genes that are enriched for the extent of p53 phosphorylation at S46 of bound p53 only upon Etoposide treatment. On a genome-wide scale p53 phosphorylated at S46 DNA-binding does not predict the occurrence or magnitude of expression change of the respective target genes. However, we did identify a group of target genes enriched for chromatin bound p53 that is phosphorylated at S46 only upon Etoposide treatment, which do change in expression accordingly. Importantly, this group includes several apoptosis related genes, like BCL2L1. This further extends the results of previous studies showing that p53 association with apoptotic target genes is affected by modifications of p53 at Serine 46 [13], [33], [34]. Our findings indicate that the Etoposide treatment which results in an increase of apoptotic cells leads to an enrichment of p53 phosphorylated at S46. This phosphorylated form of p53 can be bound to certain target genes involved in apoptosis induction. Furthermore, based on the global, genome-wide data this does not seem to be the sole functional role of p53 phosphorylation at S46. Future research using the resource of targets discovered in this study should expand the functions for which the phosphorylation of p53 at Serine 46 plays an important role.

Thus, the finding that differential binding of p53 phosphorylated at S46 to a specific subset of target genes might influence the p53 target gene choice is most likely one of several regulation layers that are required to mediate the different cellular stress response. Other modifications of p53 like acetylation of Lysine 320 could be important for the apoptotic response as well [37]. Possibly, crosstalk of several p53 modifications is necessary to induce the apoptotic stress response. For example, CBP mediated acetylation of Lysine 382 requires phosphorylation of Serine 46 [14]. Furthermore, the complexity of post-translational modifications of p53 needs to be taken into consideration to reveal the functional significance of the single modifications as well as the sequential interplay of different modifications [38]. Some of the sites for post-translational modifications are also suggested to be redundant for the p53 regulation, which makes e.g. the interpretation of knock-in studies in mice more complex [39]. Thus, future research will have to focus on the interdependent network of post-translational modifications of p53.

Besides post-translational modifications, chromatin remodeling is also involved in the target gene selectivity of p53 target genes. Recently, the human cellular apoptosis susceptibility protein (hCAS/CSE1L) was described to be involved in changing the chromatin landscape of p53 target genes [9]. hCas associates with some p53-target genes and supposedly down-regulates the methylation levels of histone H3K27, a chromatin mark which is associated with repression of transcription and heterochromatin formation. Many p53-binding proteins have been described to influence the p53-target gene choice as well. The apoptosis-stimulating proteins of p53 (ASPP) are p53-binding proteins interacting with p53 and specifically modulating p53-induced apoptosis but not cell cycle arrest [7]. Another p53-binding protein is Pin1, which upon DNA damage induced phosphorylation of p53, binds to p53 and mediates a conformational change of p53 resulting in enhanced p53 transcriptional activity [40]. Although the recruitment of p53-binding proteins, co-factors and chromatin remodelers is necessary for most p53 bound target genes to achieve gene activation or repression, Braastad et al. showed that for some p53 target genes, like p21, the chromatin seems to be in a constitutively open state, in which no extensive chromatin remodeling by co-factors is required for transcriptional regulation [41]. Since PTMs and co-factors are both involved in p53 target gene activation, there is a possibility that there is also cross talk between PTMs and co-factors. Future research should elucidate how p53 PTMs influence the interaction with co-factors and what result this has on p53 target gene selectivity. Possibly, co-factor interactions are regulated by PTMs of p53 and/or interacting proteins promote changes in p53 PTMs to achieve promoter specific transactivation.

Furthermore, regulation of target gene expression is likely also mediated by miRNAs. At the moment the involvement of these miRNAs in the decision between cell cycle arrest and apoptosis is intensively studied [42], [43], [44], [45]. In our study, we analyzed expression changes of p53 bound target genes using Affymetrix human exon arrays. Since these arrays do not contain miRNAs, we were not able to detect additional p53 responsive miRNA clusters. Thus, in the future the role of additional miRNAs on p53 target gene selectivity has to be elucidated.

In conclusion, our results suggest that in general the differential responses to Actinomycin D and Etoposide treatment cannot be explained by the overall similar p53 DNA-binding patterns. Rather, we show here that upon Etoposide treatment the DNA-bound p53 is phosphorylated at Serine 46 to a higher extent, showing that this treatment increases the DNA-bound p53 that is phosphorylated at this specific residue. Thus, in the here studied cellular system a selective context model is the most likely model to explain how p53 directs cellular outcome, since additional factors like the here shown p53-phosphorylation as well as most likely other PTMs, co-factors and chromatin remodeling influence the selectivity of target gene transactivation and therefore the cellular response pathways.

## Materials and Methods

### Cell culture and drug treatment

The human osteosarcoma cell line U2OS expressing endogenous wild-type p53, a kind gift of Karen Vousden [46], was maintained in Dulbecco modified Eagle medium supplemented with 10% fetal calf serum at 37°C. The U2OS cells were treated with 5 nM Actinomycin D (Sigma) or 10 µM Etoposide (Sigma) for 24 hours.

### Cell cycle analysis

Cells were treated as described above. The cells were fixed with 96% ethanol and stained with propidium iodide (Sigma). DNA content was analyzed by flow cytometry (Becton Dickinson FACScan) and analyzed using CellQuest Pro software.

### Immunoblotting

To assess protein levels, proteins from whole-cell extracts were harvested, lysed and separated by SDS-PAGE and analyzed by Western blotting with α-p53 (DO1, BD PharMingen), α-p53-pS15 (Cell Signaling), α-p53-pS46 (BD PharMingen), α-p21 (Ab1, Calbiochem), α-PARP (Cell signaling), α-caspase-3 (Abcam) or α-TBP (Diagenode).

### ChIP

Chromatin immunoprecipitation (ChIP) was essentially performed as described by Denissov et al. [47]. The cells were sonicated using a Bioruptor sonicator (Diagenode) for 15 minutes at high power, 30 seconds ON, 30 seconds OFF. Antibody incubation with chromatin from U2OS treated with Actinomycin D or Etoposide was performed overnight at 4°C with 1–2 µg antibody DO1 (BD PharMingen), p53-pS15 (Cell Signaling), p53-pS46 (BD PharMingen) or normal mouse IgG (Santa Cruz Biotechnology). Real-time PCR was performed using the SYBR Green mix (Biorad) with the MyIQ machine (Biorad). Primers used for real-time PCR are available upon request.

### Illumina high throughput sequencing

Sequencing samples were prepared according to the manufacturers protocol (Illumina). Shortly, adapted sequences were linked to the generated ChIP, the library was size selected (200–250 bp) and amplified by PCR. Cluster generation and 36-cycle sequencing were performed using an Illumina Cluster station and Genome Analyzer according to the manufacturer's instructions. Images acquired from the Genome Analyzer were processed through the bundled image extraction pipeline (Illumina). All 32-bp sequence reads were uniquely mapped to the human genome NCBI build 36.1 (hg18) with zero or one mismatch allowed using ELAND software (Illumina). The reads were directionally extended to 133 bp, corresponding to the length of the original fragments used for sequencing. For each position in the genome the number of overlapping sequence reads was determined, averaged over a 10 bp window and visualized in the UCSC genome browser (http://genome.ucsc.edu).

### ChIP-seq data analysis

The mapped reads were normalized between samples by uniformly removing reads to obtain an identical number of mapped reads for each experiment. Peak recognition was performed using Model-based analysis of ChIP-Seq (MACS) [21] with a p-value threshold of 1e-7. Artifacts, like spurious peaks in centromeric and telomeric regions, were removed from the list. For p53-pS15 and p53-pS46 the binding sites bound by p53 in either Actinomycin D or Etoposide treatment were analyzed. Peaks were mapped to RefSeq genes [22], downloaded from the UCSC Genome Browser, to determine genomic location. The location and score of the p53 motif within the 200 bp peaks was determined using p53scan with default settings (www.ncmls.nl/bioinfo/p53scan/) [23].

### RNA isolation and RT-PCR

Total RNA was extracted using the RNeasy Mini kit according to protocol (Qiagen). For cDNA synthesis, reverse transcription was performed with 1 µg of the total RNA, oligodT anchor primers, dNTPS, DTT, buffer and Superscript Retrotranscriptase (Invitrogen). cDNA was analyzed by qPCR using a MyIQ machine (Biorad). Primers used for real-time PCR are available upon request.

### Microarray hybridization and expression analysis

RNA from U2OS cells untreated or treated 24 hours with Actinomycin D or Etoposide was extracted using the RNeasy Mini kit according to protocol (Qiagen). A DNase I (Qiagen) digestion step was included to eliminate DNA contamination. Quantity and quality was determined by Nanodrop ND1000 and Agilent Bioanalyzer 2100. Two microgram of total RNA was used for hybridizations on the Gene Chip Human Exon 1.0 ST Array (Affymetrix) according to the manufacturer's protocol (Gene Chip® Whole Transcript (WT) Sense Target Labeling Assay Manual). Subsequently the cell-files were imported into Partek® (Partek® Genomic Suite software, version 6.4 Copyright © 2008 Partek Inc., St. Louis, MO, USA) where only core exons were extracted and normalized using the Robust Multiarray Analysis (RMA) [48] algorithm with GC background correction. Core transcript summaries were calculated using the mean intensities of the corresponding probe sets [49]. All samples were performed in duplicates. An average expression value for each time point (0 and 24 hours) was obtained by averaging the median normalized gene-level expression value for the two replicate per time point. The expression values for 24 hours were divided by the expression values of 0 hours resulting in a ratio for each gene. Affymetrix core transcripts with a p53-binding site within the transcript or 25 kb up- or downstream were clustered into 10 groups with Cluster3 [50] using the k-Means algorithm based on uncentered correlation. Results were visualized with Java TreeView [51].

### Data availability

The data have been deposited in NCBI's Gene Expression Omnibus [52] and are accessible through GEO Series accession number GSE22186 (http://www.ncbi.nlm.nih.gov/geo/query/acc.cgi?acc=GSE22186).

## Supporting Information

Figure S1 **Characterization of preferential peaks.** (A) ChIP recovery of p53-binding to the p21 and BAX promoter compared to IgG as a negative control in U2OS cells treated for 24 hours with 5 nM Actinomycin D (left panel) or 10 µM Etoposide (right panel). ChIP was performed with a p53-antibody (p53-DO1) or an IgG antibody and qPCR analysis was performed with primers for the respective binding sites. Binding to Myoglobin (myo) was used as a negative control. Error bars represent standard deviation of three individual experiments. (B) The average number of reads per peak for the preferential p53 peaks as well as random reads upon Actinomycin D treatment (left panel) or Etoposide treatment (right panel) are visualized in a boxplot.
(TIF)

Figure S2 **Correlation between p53-binding and expression change.** Change in expression ratio was plotted against the p53 number of reads per peak (RPP) of the ChIP-Seq experiment for Actinomycin D (left panel) and Etoposide (right panel) treatment.
(TIF)

Figure S3 **Binding overlap between different experiments.** Overlap op p53, p53-pS15 and p53-pS46 binding as determined by ChIP-Seq in U2OS cells treated with Actinomycin D (left panel) or Etoposide (right panel) for 24 hours.
(TIF)

Figure S4 **Binding of phosphorylated p53 to apoptotic and growth arrest target genes.** p53, p53-pS15 and p53-pS46 binding as determined by ChIP-Seq with the Genome analyzer (Illumina) and visualized using the UCSC genome browser. Shown are binding loci of the apoptotic target genes BAX and PUMA, and binding loci of two growth arrest target genes p21 and MDM2 of Actinomycin D treated cells (left panel) and Etoposide treated cells (right panel).
(TIF)

Figure S5 **P53-pS15 binding to selectively bound p53-pS46 target genes.** ChIP-qPCR recovery of p53-pS15 at loci which show a higher degree of p53 phosphorylated at S46 upon Etoposide treatment. U2OS cells were treated with Actinomycin D or Etoposide for 24 hours, before chromatin was isolated. ChIP was performed with p53-pS15-antibody and qPCR analysis was performed with primers for the putative binding sites. Shown is the recovery of p53-pS15 normalized to the recovery of total p53-DO1 binding in Etoposide or Actinomycin D treated U2OS-cells. Error bars represent standard deviation of three individual experiments.
(TIF)

Table S1 (XLS)

Table S2 (XLS)

Table S3 (XLS)

Table S4 (XLS)
