# Supplementary figures and images for: Role of p53 Serine 46 in p53 Target Gene Regulation
Source: PLoS One. 2011 Mar 4;6(3):e17574. doi: 10.1371/journal.pone.0017574 (PMC3048874; doi:10.1371/journal.pone.0017574)

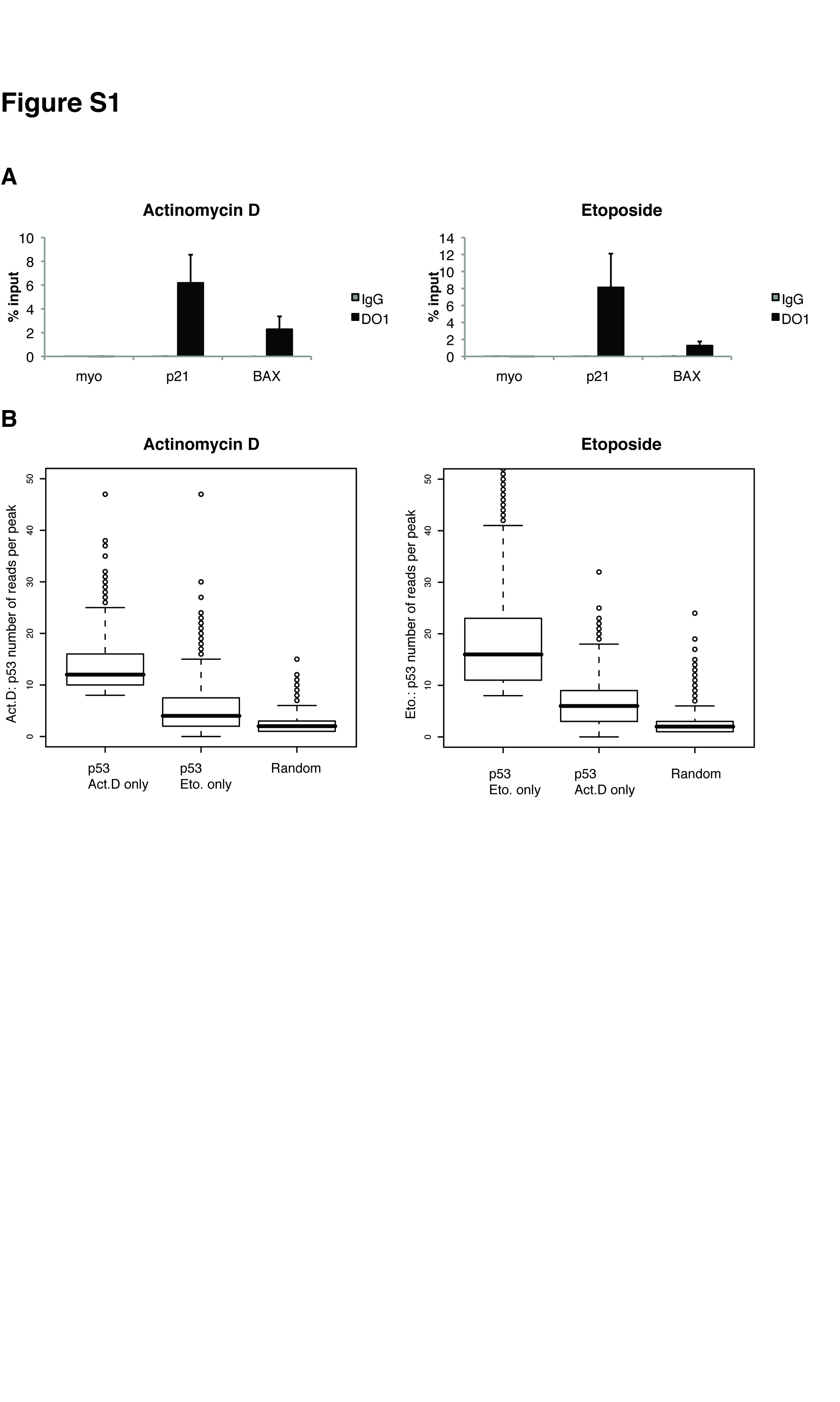

Supplement: Figure S1 — Characterization of preferential peaks. (A) ChIP recovery of p53-binding to the p21 and BAX promoter compared to IgG as a negative control in U2OS cells treated for 24 hours with 5 nM Actinomycin D (left panel) or 10 µM Etoposide (right panel). ChIP was performed with a p53-antibody (p53-DO1) or an IgG antibody and qPCR analysis was performed with primers for the respective binding sites. Binding to Myoglobin (myo) was used as a negative control. Error bars represent standard deviation of three individual experiments. (B) The average number of reads per peak for the preferential p53 peaks as well as random reads upon Actinomycin D treatment (left panel) or Etoposide treatment (right panel) are visualized in a boxplot. (TIF) [file pone.0017574.s001.tif]

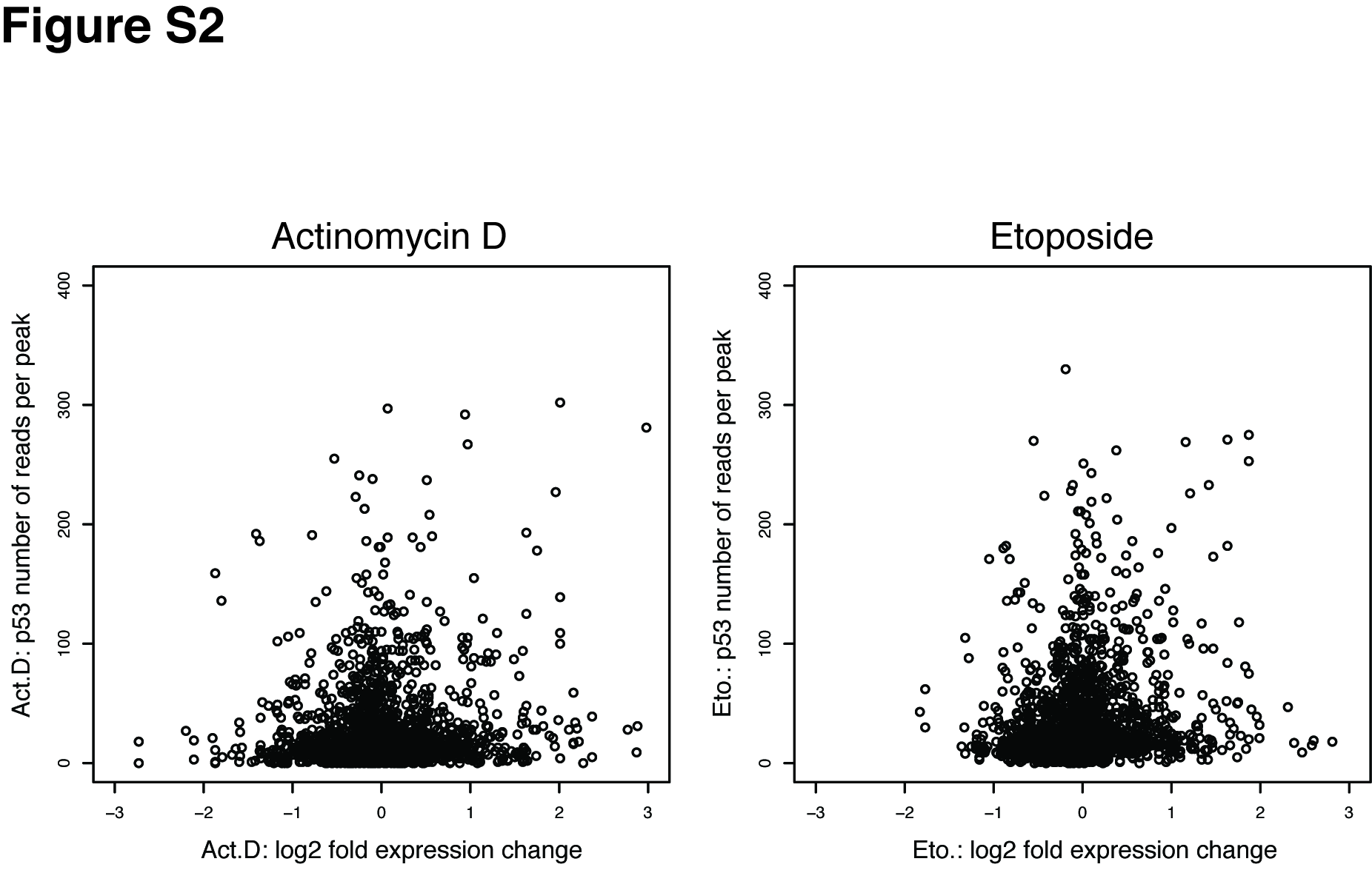

Supplement: Figure S2 — Correlation between p53-binding and expression change. Change in expression ratio was plotted against the p53 number of reads per peak (RPP) of the ChIP-Seq experiment for Actinomycin D (left panel) and Etoposide (right panel) treatment. (TIF) [file pone.0017574.s002.tif]

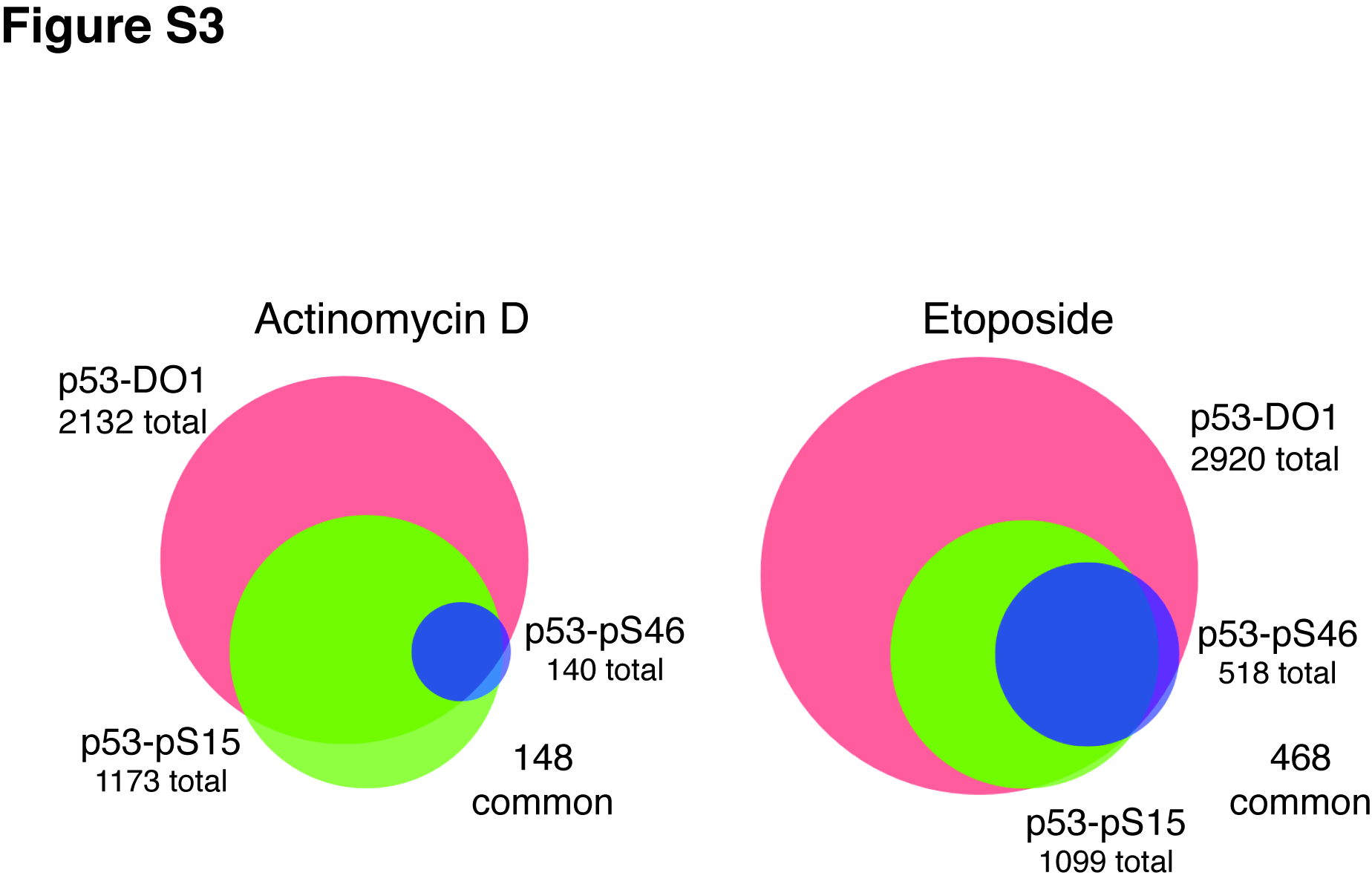

Supplement: Figure S3 — Binding overlap between different experiments. Overlap op p53, p53-pS15 and p53-pS46 binding as determined by ChIP-Seq in U2OS cells treated with Actinomycin D (left panel) or Etoposide (right panel) for 24 hours. (TIF) [file pone.0017574.s003.tif]

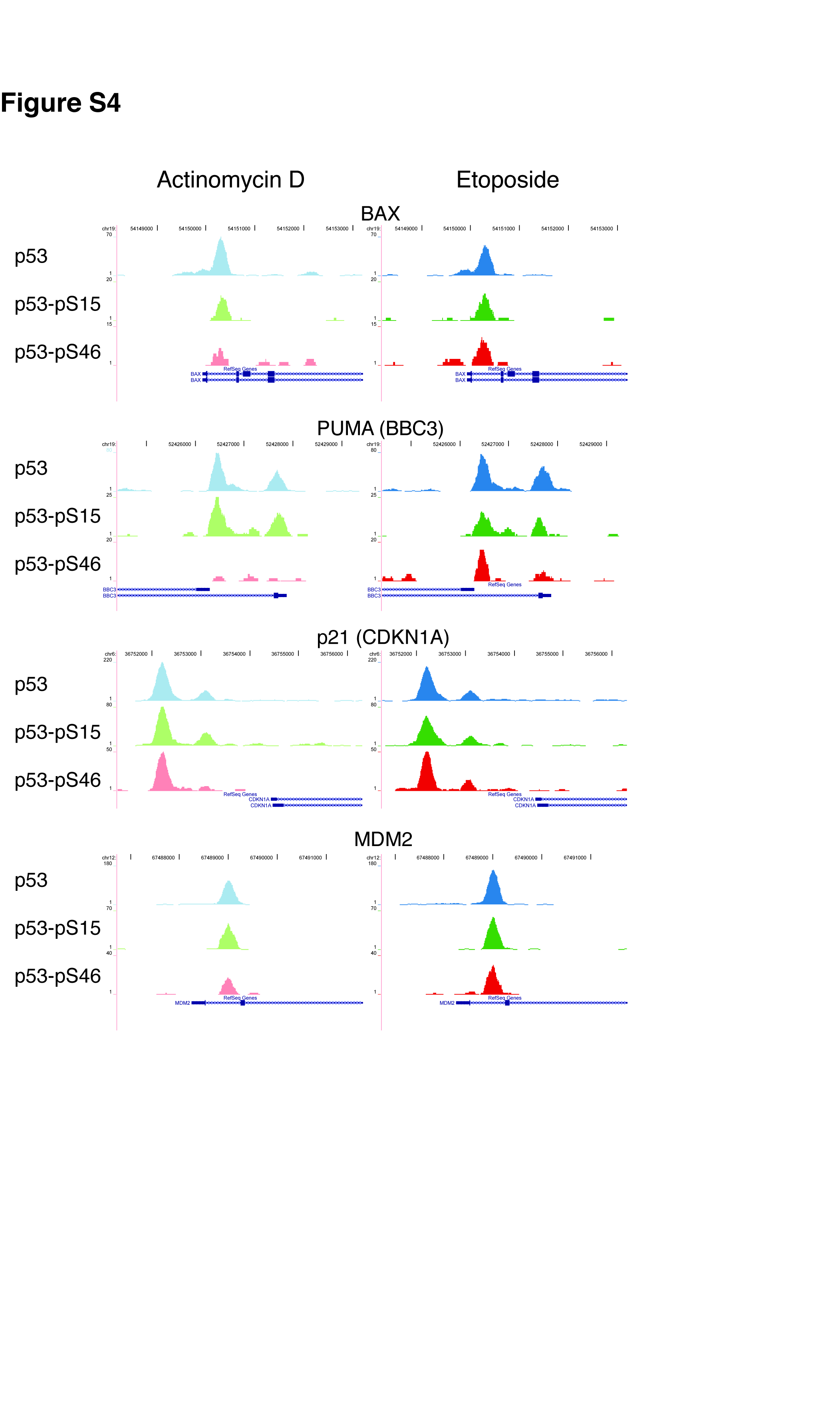

Supplement: Figure S4 — Binding of phosphorylated p53 to apoptotic and growth arrest target genes. p53, p53-pS15 and p53-pS46 binding as determined by ChIP-Seq with the Genome analyzer (Illumina) and visualized using the UCSC genome browser. Shown are binding loci of the apoptotic target genes BAX and PUMA, and binding loci of two growth arrest target genes p21 and MDM2 of Actinomycin D treated cells (left panel) and Etoposide treated cells (right panel). (TIF) [file pone.0017574.s004.tif]

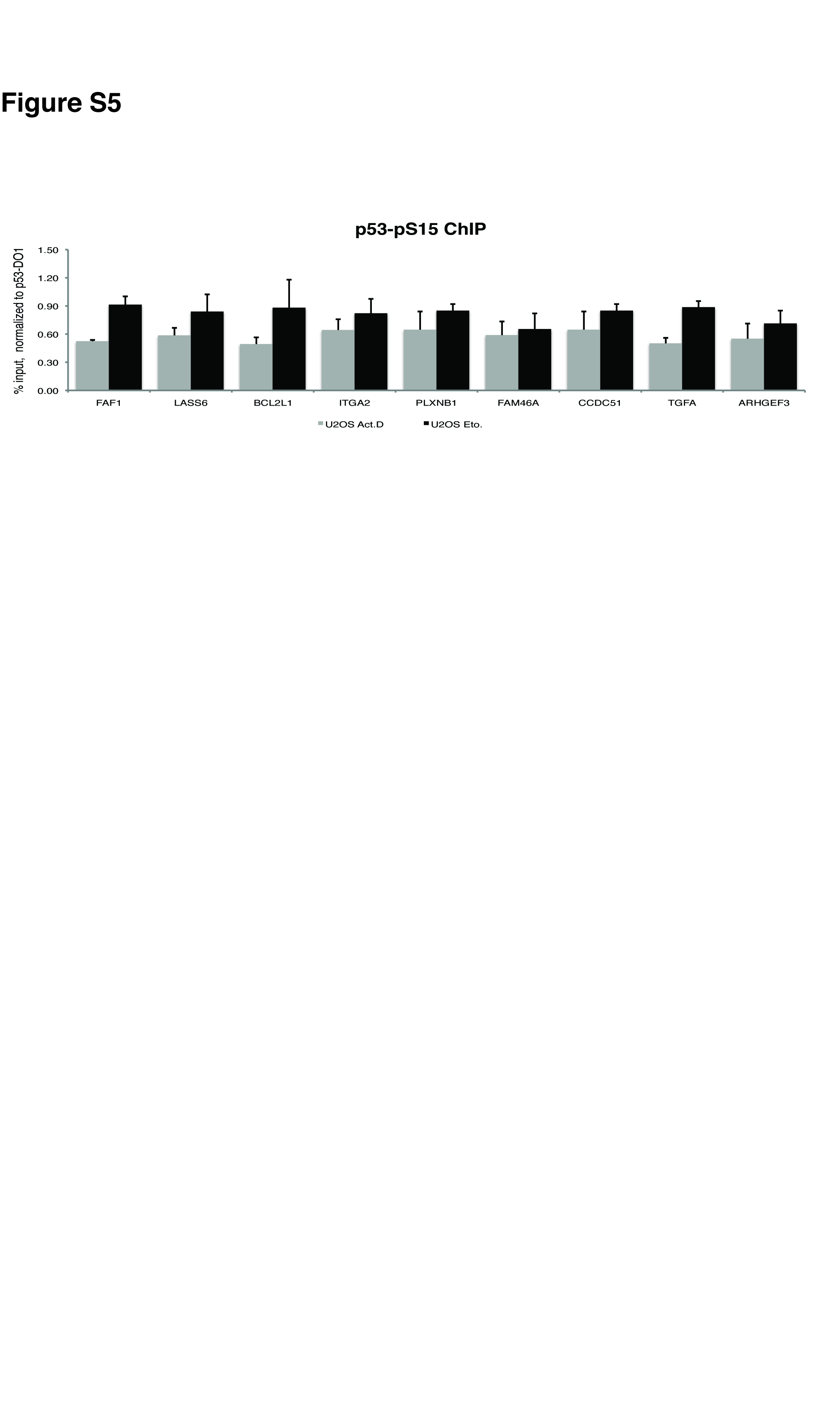

Supplement: Figure S5 — P53-pS15 binding to selectively bound p53-pS46 target genes. ChIP-qPCR recovery of p53-pS15 at loci which show a higher degree of p53 phosphorylated at S46 upon Etoposide treatment. U2OS cells were treated with Actinomycin D or Etoposide for 24 hours, before chromatin was isolated. ChIP was performed with p53-pS15-antibody and qPCR analysis was performed with primers for the putative binding sites. Shown is the recovery of p53-pS15 normalized to the recovery of total p53-DO1 binding in Etoposide or Actinomycin D treated U2OS-cells. Error bars represent standard deviation of three individual experiments. (TIF) [file pone.0017574.s005.tif]
